# The Hierarchy of Exon-Junction Complex Assembly by the Spliceosome Explains Key Features of Mammalian Nonsense-Mediated mRNA Decay

**DOI:** 10.1371/journal.pbio.1000120

**Published:** 2009-05-26

**Authors:** Niels H. Gehring, Styliani Lamprinaki, Matthias W. Hentze, Andreas E. Kulozik

**Affiliations:** 1 Molecular Medicine Partnership Unit, University of Heidelberg and European Molecular Biology Laboratory, Heidelberg, Germany; 2 Department of Pediatric Oncology, Hematology and Immunology, University of Heidelberg, Heidelberg, Germany; 3 European Molecular Biology Laboratory, Heidelberg, Germany; National Cancer Institute, United States of America

## Abstract

Exon junction complexes (EJCs) link nuclear splicing to key features of mRNA function including mRNA stability, translation, and localization. We analyzed the formation of EJCs by the spliceosome, the physiological EJC assembly machinery. We studied a comprehensive set of eIF4A3, MAGOH, and BTZ mutants in complete or C-complex–arrested splicing reactions and identified essential interactions of EJC proteins during and after EJC assembly. These data establish that EJC deposition proceeds through a defined intermediate, the pre-EJC, as an ordered, sequential process that is coordinated by splicing. The pre-EJC consists of eIF4A3 and MAGOH-Y14, is formed before exon ligation, and provides a binding platform for peripheral EJC components that join after release from the spliceosome and connect the core structure with function. Specifically, we identified BTZ to bridge the EJC to the nonsense-mediated messenger RNA (mRNA) decay protein UPF1, uncovering a critical link between mRNP architecture and mRNA stability. Based on this systematic analysis of EJC assembly by the spliceosome, we propose a model of how a functional EJC is assembled in a strictly sequential and hierarchical fashion, including nuclear splicing-dependent and cytoplasmic steps.

## Abstract 2

Protein complexes deposited on messenger RNAs during their maturation are able to recruit components of a cellular RNA surveillance pathway, thereby linking RNA maturation to subsequent steps in RNA quality control.

## Author Summary

The first step in the expression of eukaryotic protein-coding genes is transcription into a messenger RNA (mRNA) precursor in the nucleus. These precursor mRNAs then undergo maturation through the removal of introns in a process termed splicing. During splicing, the splicing machinery or “spliceosome” deposits a complex of proteins onto the mRNA that accompanies it during post-transcriptional steps in gene expression, including the regulation of mRNA stability, transport out of the nucleus, cellular localisation, and translation. This complex, the exon junction complex (EJC), represents a molecular memory of the splicing process. Understanding the biogenesis of EJCs and their downstream effects helps reveal the basic principles by which the primary steps of mRNA synthesis are coupled to the regulation of gene expression. Here we show that EJCs are assembled in a strictly splicing-dependent manner through an unexpected, coordinated, and hierarchical assembly pathway. Importantly, we show that the EJC recruits the cytoplasmic protein BTZ, which then bridges the complex to an mRNA quality-control machinery called the nonsense-mediated decay pathway that degrades mRNAs containing premature stop codons. This finding suggests that the EJC and bridging by BTZ help determine the stability of mRNA and thus are essential for proper cellular surveillance of mRNA quality.

## Introduction

Gene expression in eukaryotes involves multiple post-transcriptional steps, including pre–messenger RNA (mRNA) processing, the export of the mature mRNA to the cytoplasm, its correct intracellular localization, and finally its translation and turnover [1],[2]. All these processes are coordinated by a network of communicating cellular machines [3]. The exon junction complex (EJC) plays a central role in the coordination of post-transcriptional gene expression in metazoan cells. The EJC is deposited on nascent mRNAs during splicing in a sequence-independent manner 20–24 nucleotides (nts) upstream of exon–exon junctions [4]. EJCs communicate the pre-splicing architecture of a spliced mRNA to cytoplasmic processes and modulate central events in gene expression such as nuclear mRNA export, mRNA quality control by nonsense-mediated mRNA decay (NMD), and translation of mRNAs in the cytoplasm [5]–[7].

NMD represents an intensively studied splicing- and translation-dependent process that limits the expression of abnormal transcripts containing premature termination codons and controls the expression of normal mRNA isoforms that are generated from the same pre-mRNA at different times of development and in different tissues [8]–[11]. As such, NMD has broad biological and medical implications [12]. NMD can be recapitulated by introducing a functional intron into the 3′ untranslated region (UTR) of an otherwise wild-type mRNA or by tethering either of the EJC components MAGOH, Y14, eIF4A3 (DDX48), or Barentsz (BTZ, also referred to as MLN51 or CASC3) to the 3′ UTR of reporter mRNAs in human cells [13]–[17]. These data indicate that the presence of an EJC at an appropriate distance downstream of a termination codon is sufficient to elicit NMD and suggest that the EJC provides the direct molecular link for the recognition of premature translation termination codons.

The core of the EJC, consisting of the four proteins eIF4A3, MAGOH, Y14, and BTZ, can be assembled from recombinant subunits in vitro when its components are simultaneously present [18]. Such in vitro assembly of the EJC core also requires the presence of ATP and single-stranded RNA, both of which are an integral part of the complex [18]. The crystal structure of this core EJC bound to oligo-U RNA shows that eIF4A3 binds the phosphate–sugar backbone of the RNA via its DEAD-box helicase domain. This structure explains why the binding of RNA by the EJC is stable and specific for RNA, despite being sequence-independent [19],[20]. Binding of RNA requires simultaneous binding of a molecule of ATP, whereas ATP hydrolysis by eIF4A3's inherent ATPase activity leads to the dissociation of the EJC from the RNA [18]. To stably clamp the EJC on the RNA, the ATPase activity of eIF4A3 is inhibited by the binding of the MAGOH-Y14 heterodimer to eIF4A3 [18]. Interestingly, eIF4A3 can undergo a remarkable structural reorganization [19],[20]. Whereas BTZ binds eIF4A3 in both the open and the closed RNA-bound conformations, MAGOH-Y14 binding to eIF4A3 occurs only in its RNA-bound state.

The protein PYM also plays an important role both in the function and in the recycling of EJCs. Ribosome-associated PYM binds to the EJC components MAGOH-Y14 [21], thereby recruiting ribosomes to spliced mRNAs to stimulate translation as well as directing the disassembly of EJCs in the cytoplasm [22],[23].

The determination of the crystal structure of the biochemically assembled EJC core has been a milestone in the analysis of EJC function. However, a better biological understanding of EJC assembly will have to integrate this structural information with the fact that EJC assembly in vivo is strictly splicing-dependent [4],[24],[25]. We have thus investigated spliceosome-dependent EJC assembly and demonstrate that the EJC is assembled along a defined hierarchical pathway that ensures the proper positioning and stable binding of the EJC on the (pre-)mRNA substrate. Our data define a stable minimal pre-EJC core consisting of eIF4A3 and MAGOH-Y14, which serves as a binding platform for EJC binding factors like BTZ and UPF3b that link the EJC to functional downstream effectors.

## Results

### Splicing-Dependent EJC Assembly In Vitro

**Figure 1:**
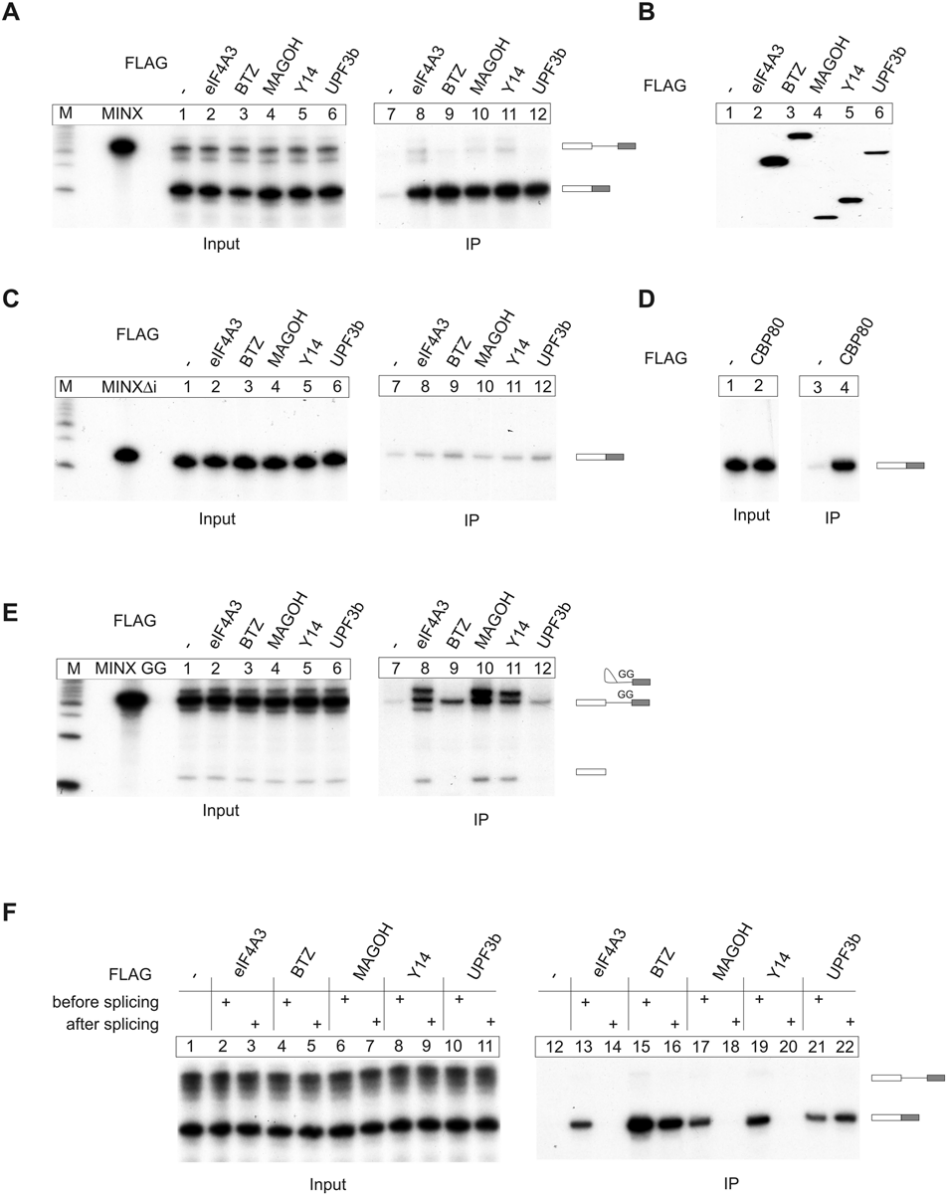
Ordered, spliceosome-mediated assembly of the EJC. (A) Splicing reactions using MINX as substrate RNA were supplemented with extracts expressing the indicated FLAG-tagged EJC proteins or unfused FLAG-tag as negative control. Reactions were immunoprecipitated with FLAG affinity gel. Positions of the unspliced transcript and the spliced product are displayed schematically. Twelve percent of the total input material was loaded in the input panel. (B) Expression of the FLAG-proteins used in (A) determined by immunoblot analysis with a FLAG antibody. (C) Splicing reactions and immunoprecipitations were performed as in (A) with an intronless MINX transcript (MINX Δi). (D) Splicing reactions and immunoprecipitations with FLAG-CBP80 or unfused FLAG employing the intronless MINX transcript (MINX Δi) used in (C). (E) Splicing reactions and immunoprecipitations were performed as in (A) with a mutated MINX transcript (MINX GG) that does not undergo exon ligation. Positions of the unspliced transcript and the splicing intermediates (exon 1; lariat and exon 2) are displayed schematically. 8% of the total input material was loaded in the input panel. (F) Splicing reactions were performed in the presence (lanes 2, 4, 6, 8, 10, 13, 15, 17, 19, and 21) or absence (lanes 3, 5, 7, 9, 11, 14, 16, 18, 20, and 22) of FLAG-protein extracts or with unfused FLAG as negative control (lanes 1 and 12). Reactions without added FLAG-proteins were supplemented after the completion of splicing with FLAG-protein extracts and incubated on ice (30 min). Immunoprecipitations were done as in (A), except that 15% of the total input material was loaded in the input panel.

We first established an experimental system to investigate the splicing-dependent deposition of EJC components with MINX pre-mRNA that contains an intron that activates NMD when introduced into the 3′ UTR of a β-globin mRNA [17]. HeLa nuclear extract was supplemented with splicing-competent whole-cell extracts from HEK293 cells expressing different FLAG-tagged EJC proteins (Figure 1B). When MINX transcripts are incubated under splicing conditions in such mixed HeLa-HEK293 extracts, they are spliced efficiently and processed to mRNAs of the expected size (Figure 1A). From these splicing reactions, the FLAG-tagged EJC proteins are immunoprecipitated together with their associated mRNAs. As expected for EJC proteins, the fully spliced mRNAs specifically co-immunoprecipitate with FLAG-eIF4A3, -BTZ, -MAGOH, -Y14, and -UPF3b. In contrast, only small amounts of splicing intermediates or pre-mRNAs associate with FLAG-eIF4A3, -MAGOH, -Y14, or BTZ (Figure 1A). These immunoprecipitations are specific for spliced RNAs, as only traces of an intronless MINX transcript (MINX Δi) are pulled down under the same conditions (Figure 1C). As expected, the intronless transcript co-immunoprecipitates with FLAG-CBP80 (Figure 1D). This protein associates with mRNAs in a splicing-independent but cap-dependent manner, and hence serves as a positive control for the overall integrity and protein binding capacity of the MINX Δi RNA.

### eIF4A3 and MAGOH-Y14 Form a Pre-EJC before Exon Ligation

Purified spliceosomes, as well as purified spliceosomal C-complexes, contain the EJC proteins eIF4A3, Y14, and MAGOH [26]–[28]. This poses the question of when and in which order individual EJC proteins are recruited to the RNA during splicing. To answer this question, we performed FLAG immunoprecipitations from splicing reactions using a MINX pre-mRNA with a GG mutation of the 3′ splice site. This manipulation completely blocks splicing after intron lariat formation and before exon ligation, and also noticeably decreases the efficiency of the first step of splicing [29],[30]. The splicing intermediates (intron lariat-3′ exon, 5′ exon) and pre-mRNA are specifically precipitated via eIF4A3, MAGOH, and Y14 (Figure 1E, lanes 8, 10, and 11). Whereas UPF3b precipitates the pre-mRNA only marginally above background and fails to precipitate splicing intermediates, BTZ precipitates some pre-mRNA and also no intermediates (Figure 1E, lanes 9 and 12). BTZ may thus interact with the pre-mRNA under these conditions, but is not detectable in the lariat mRNP. This finding implies that BTZ and UPF3b appear not to associate with the RNA at an early step of splicing, but are rather recruited at later stages or after splicing of the mRNA has been completed. This result was expected for UPF3b and confirms the current view that UPF3b binds only to the fully assembled EJC after release of the spliceosome. In contrast, we were surprised to discover that BTZ does not associate with eIF4A3 during splicing and may even be actively excluded from the spliceosomal C-complex (see below), because BTZ can interact with eIF4A3 in vitro even in the absence of Y14 and MAGOH [20],[31].

These results show that the biochemically defined EJC core is assembled by the spliceosome in a stepwise manner. The three proteins eIF4A3, MAGOH, and Y14 first assemble on the RNA to form a pre-EJC, which is subsequently bound by BTZ (and UPF3b) to yield the core EJC. This assembly hierarchy predicts either that BTZ binds to eIF4A3 during the second step of splicing (after the minimal trimeric pre-EJC is fully assembled), or that binding occurs when the spliceosome has dissociated from the mRNA. To distinguish between these two alternatives, splicing reactions were performed in extracts with or without the initial addition of HEK293 cell extracts expressing the FLAG-tagged EJC proteins (Figure 1F). Reactions that initially lacked FLAG-tagged proteins were supplemented with the same amount of FLAG-extracts after the completion of splicing and chased on ice to monitor the exchange of EJC-bound factors with supplemented proteins. Afterwards, recombinant proteins and associated mRNAs were affinity selected by FLAG immunoprecipitation. The EJC proteins eIF4A3, MAGOH, and Y14 co-purified with spliced mRNA only if they were present before the beginning of splicing (Figure 1F, lanes 13, 14, 17–20). This result is expected for factors that bind to the mRNA in a splicing-dependent manner with little exchange of components after EJC assembly and the completion of splicing. Interestingly, BTZ and UPF3b co-purify with the spliced mRNA independent of whether they were added before or after splicing (Figure 1F). Thus, BTZ and UPF3b bind to the minimal trimeric pre-EJC that is loaded onto the RNA in a splicing-dependent manner, but themselves do not require splicing for EJC binding. Similar results were obtained when splicing reactions were supplemented with recombinant proteins instead of HEK293 cell extracts (Figure S1).

### MAGOH-Y14 Requires eIF4A3 for EJC Binding

We next defined the functions of the individual EJC proteins during the assembly of the EJC as well as their roles in NMD. Considering the published crystal structures of the MAGOH-Y14 heterodimer [13],[32],[33], the MAGOH-Y14-PYM trimer [21], and the EJC [19],[20], we used mutants of MAGOH, eIF4A3, and BTZ that were designed to specifically disrupt important interaction sites.

**Figure 2:**
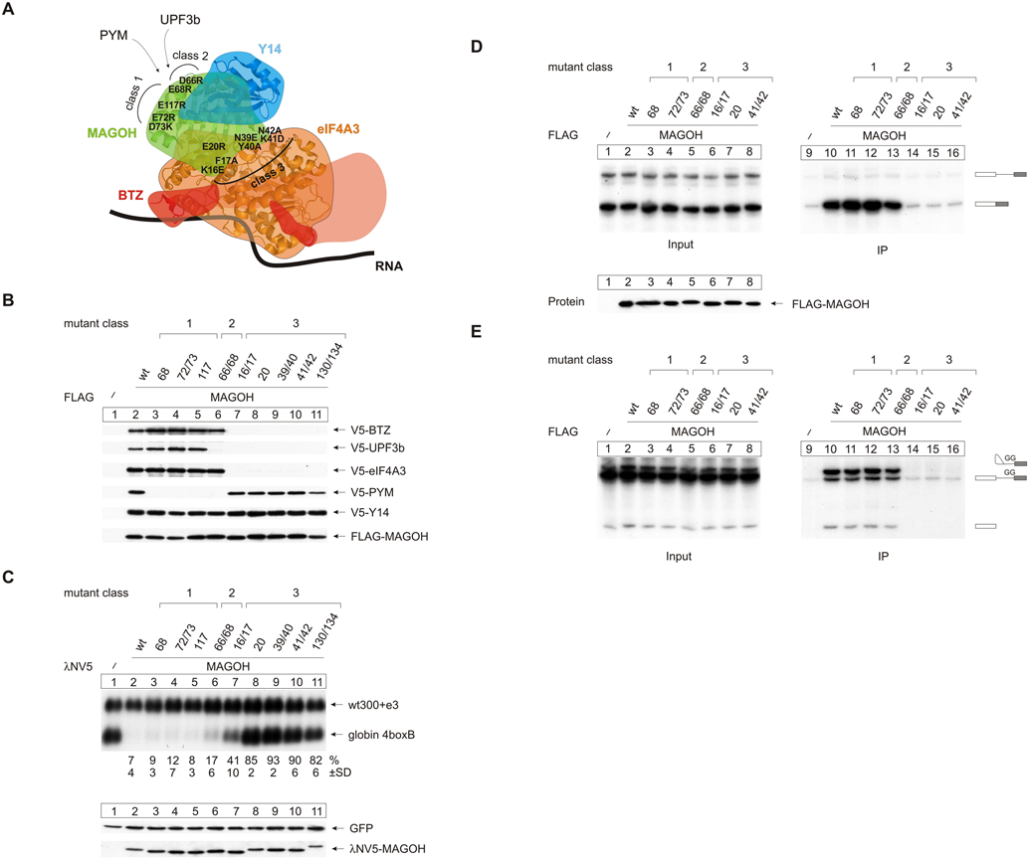
MAGOH-Y14 are recruited to the EJC by eIF4A3. (A) Scheme of the positions of the MAGOH mutants in the EJC. The interactions with UPF3b and PYM are indicated by arrows. BTZ residues present in the crystal structure are displayed in red, an arbitrary shape of full length BTZ is suggested in light red. The EJC structure was rendered using PyMOL [54] with structural data deposited in the Protein Data Bank (http://www.rcsb.org/pdb/home/home.do; ID: 2j0s). (B) Immunoprecipitations were done from RNAse A-treated lysates of HeLa cells that were transfected with FLAG-MAGOH and FLAG-MAGOH mutants or unfused FLAG as negative control together with V5-tagged BTZ, UPF3b, eIF4A3, PYM, and Y14. Co-precipitated proteins were detected by immunoblotting using an anti V5 antibody. (C) Northern blot analysis of RNA from HeLa cells that were transfected with expression plasmids for λNV5-tagged MAGOH or mutants of MAGOH together with the 4boxB reporter plasmid and the transfection control plasmid. Percentages (%) represent the mean of four independent experiments±standard deviations (SD). Bottom panel: expression levels of the tethered MAGOH mutant proteins were detected by immunoblot analysis with a V5-specific antibody. GFP served as internal loading control. (D) Splicing reactions using MINX as substrate RNA were supplemented with extracts expressing the indicated FLAG-tagged MAGOH mutants or unfused FLAG-tag as negative control. Reactions were immunoprecipitated with FLAG affinity gel. Twelve percent of the total input material was loaded in the input panel. (E) Splicing reactions and immunoprecipitations were performed as in (D) with the mutated MINX GG transcript.

FLAG immunoprecipitation experiments were performed to characterize protein–protein interactions of the MAGOH mutants (Figure 2A and Table S1). In agreement with previous data [14] and their position within the EJC, each mutant belongs to one of three different classes: (1) mutants that do not bind PYM (68, 72/73, 117; Figure 2B, lanes 3–5), (2) mutants that bind neither PYM nor UPF3b (66/68; Figure 2B, lane 6), or (3) mutants that do not bind eIF4A3, BTZ, and UPF3b (16/17, 20, 39/40, 41/42, 130/134; Figure 2B, lanes 7–11). For previously described MAGOH mutants, comparable co-immunoprecipitation results were obtained with endogenous Y14, PYM, eIF4A3, UPF3b, and BTZ [14], indicating that the tagged proteins reflect the function of the endogenous proteins. We next analyzed the function of the different mutants in NMD by using a tethering system. The activation of NMD in this assay is achieved by single EJC proteins tethered to the 3′ UTR of a reporter mRNA. This experimental system has served in functional analyses of mutant NMD factors and to dissect different steps of the NMD pathway(s) [14],[15],[34],[35]. These useful properties of tethering experiments must, however, be seen against the background that the physiological situation is not likely reflected in full because of the inherently artificial nature of the assay and the fact that some of the steps of EJC assembly are bypassed. In NMD tethering assays, mutants of the three groups display distinct characteristics (Figure 2C). Class 1 mutants are as active as the wild-type MAGOH (lanes 3–5), while the class 2 mutant is approximately 2-fold less active (lane 6), consistent with previously reported data [14]. Strikingly, the class 3 mutants are inactive or show a dramatically reduced NMD activity (Figure 2C, lanes 7–11). Taken together, these results suggest that tight binding of UPF3b to MAGOH is not strictly required for NMD in the context of tethered MAGOH. This finding is consistent with a report of a UPF3-independent NMD pathway [36] and the normal NMD efficiency of UPF3b-deficient cells [37]. By contrast, the recruitment of eIF4A3 and BTZ appears to be a prerequisite for efficient activation of NMD in the context of tethered MAGOH (Figure 2C, lanes 7–11).

We selected six representative MAGOH mutants from the three different classes to analyze their association with the completely spliced mRNA (Figure 2D) and with the trapped intron-lariat (Figure 2E). The different classes of mutants display distinct differences regarding EJC formation compared to wild-type MAGOH. Class 1 mutants co-immunoprecipitate slightly, but reproducibly more spliced MINX mRNA (Figure 2D, compare lanes 11 and 12 with lane 10). The class 2 mutant precipitates similar amounts of MINX RNA as wild-type MAGOH (compare lanes 10 and 13). Finally, class 3 mutants fail to associate with the spliced MINX mRNA. These results fit well with earlier data that implicate eIF4A3 as the RNA-binding platform for the other EJC proteins and that suggested that both MAGOH and Y14 associate with RNA indirectly by binding to eIF4A3 [13],[18]–[20],[38]. Additionally, these data indicate that the interaction of PYM with MAGOH-Y14 is not required for the stable assembly of the EJC. Our analyses of the lariat RNP (C-complex) with class 1 and class 2 mutants of MAGOH (Figure 2E) demonstrate that neither PYM nor UPF3b are required for loading of MAGOH onto the pre-mRNA and for the interaction of MAGOH with the C-complex (lanes 10–13). In contrast, the class 3 mutants show that MAGOH requires its eIF4A3 interaction to efficiently associate with the pre-mRNA and with the splicing intermediates of the C-complex (Figure 2E, lanes 14–16). These data suggest that MAGOH and Y14 are recruited to the RNA via their eIF4A3 interaction. This recruitment step of MAGOH-Y14 may be mediated exclusively by eIF4A3, but it could involve additional interactions of MAGOH-Y14 with spliceosomal proteins.

### eIF4A3 Is Loaded onto the mRNA by Splicing and Requires MAGOH-Y14 for Tight RNA Binding

**Figure 3 pbio-1000120-g003:**
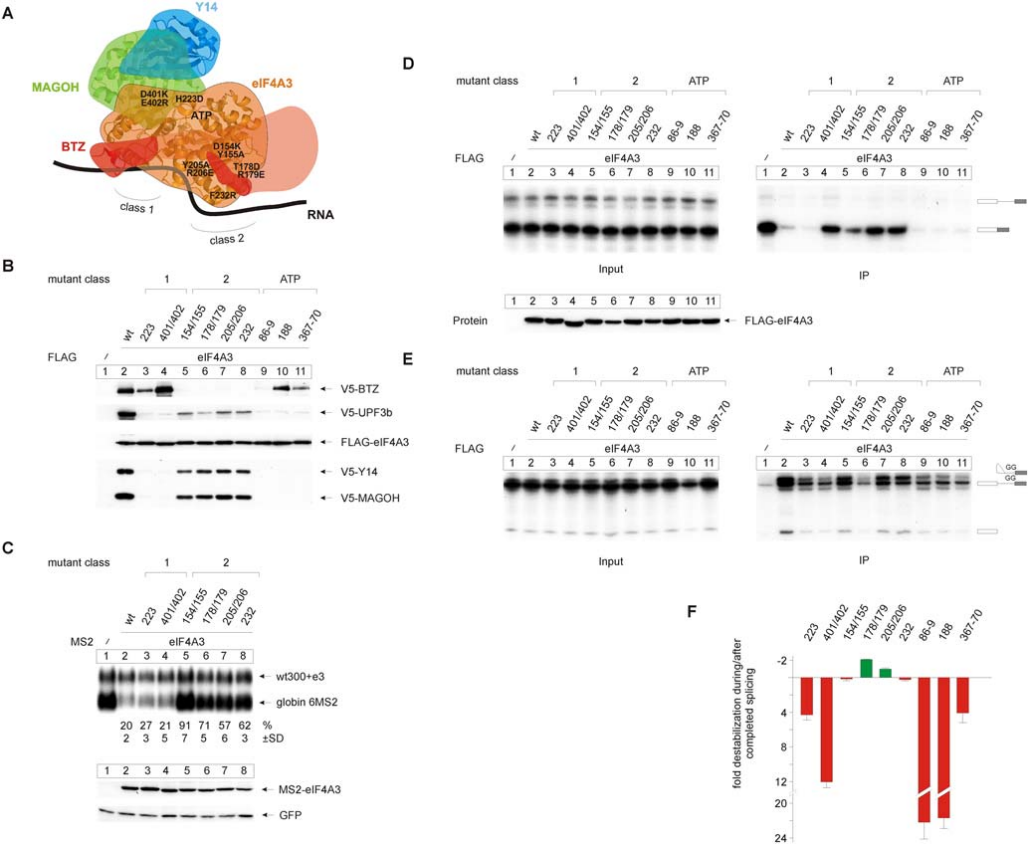
Functional analysis of eIF4A3 in EJC assembly and NMD. (A) Positions of the eIF4A3 mutants within the EJC as shown in Figure 2. Mutants in the ATP binding pocket on the inside of the protein and are not shown. (B) Immunoprecipitations were done from RNAse A-treated lysates of HeLa cells, which were transfected with FLAG-eIF4A3 and FLAG-eIF4A3 mutants or unfused FLAG as negative control together with V5-tagged BTZ, UPF3b, Y14, and MAGOH. Co-precipitated proteins were detected by immunoblotting using an anti V5 antibody. (C) Northern blot analysis of total cytoplasmic RNA from HeLa cells that were transfected with expression plasmids for MS2-tagged eIF4A3 or mutants of eIF4A3 together with the 6MS2 reporter plasmid and the transfection control plasmid. Percentages (%) represent the mean of 3 independent experiments±standard deviations (SD). Bottom panel: expression levels of the tethered eIF4A3 mutants were detected by immunoblot analysis with a MS2-specific antibody. GFP served as internal loading control. (D) Splicing reactions using MINX as substrate RNA were supplemented with extracts expressing the indicated FLAG-tagged eIF4A3 mutants or unfused FLAG-tag as negative control. Reactions were immunoprecipitated with FLAG affinity gel. Twelve percent of the total input material was loaded in the input panel. (E) Splicing reactions and immunoprecipitations were performed as in (C) with the mutated MINX GG transcript. (F) The amounts of MINX or MINX GG RNA immunoprecipitated by eIF4A3 mutants as shown in (C) and (D) were normalized to the amount precipitated by the eIF4A3 wild-type. Mutants that precipitated a lower relative amount of MINX compared to MINX GG were considered to destabilize the EJC during or after completion of splicing (red columns). Conversely, mutants that precipitated higher amounts of MINX compared to MINX GG were considered to increase the stability of the mature EJC (green columns).

Next, mutants of eIF4A3 (Figure 3A) were generated that lack binding to either MAGOH-Y14 (223, 401/402; class 1) or BTZ (154/155, 178/179, 205/206, 232; class 2). In addition, point mutations within the ATP binding pocket of eIF4A3 were generated that are expected to interfere with ATP binding (labeled ATP in Figure 3A). FLAG immunoprecipiations were performed to analyze the interaction of these mutants with BTZ, UPF3b, Y14, and MAGOH(Figure 3B). As expected, the class 1 mutants 223 and 401/402 fail to co-precipitate MAGOH-Y14 (Figure 3B, lanes 3 and 4). Similarly, only traces of UPF3b are detectable. The interaction with BTZ is not affected by the 401/402 mutation, whereas the 223 mutation decreases this interaction slightly. By contrast, the class 2 mutants 154/155, 178/179, 205/206, and 232 abolish the interaction of eIF4A3 with BTZ, while the MAGOH-Y14 interaction is maintained, albeit at a reduced level (lanes 5–8). Co-precipitation of UPF3b is also detected, albeit less than with wild-type eIF4A3 (lane 2), but considerably more than seen with the class 1 mutants. All three ATP binding mutants fail to co-precipitate MAGOH-Y14 and bind only traces of UPF3b (lanes 9–11), one mutant does not precipitate BTZ (lane 9). The two other mutants, however, co-precipitate significant amounts of BTZ (lanes 10 and 11). Hence, the binding of eIF4A3 to BTZ or MAGOH-Y14 can be separated and thus occur independently from one another. These results also indicate that UPF3b binding to the EJC requires a heterotrimer consisting of eIF4A3, MAGOH, and Y14 and is likely supported by BTZ. Binding of MAGOH-Y14 occurs apparently only in the ATP-bound closed conformation, whereas BTZ binding occurs independently of ATP binding.

We next tested the class 1 and the class 2 eIF4A3 mutants in the NMD tethering assay. Surprisingly, MAGOH-Y14 binding (and hence tight UPF3b binding) is not required for NMD activity of tethered eIF4A3 (Figure 3C, lanes 3 and 4). In contrast, tethered eIF4A3 shows markedly reduced or no NMD activity when BTZ binding is abolished (Figure 3C, lanes 5–8). This finding nicely complements earlier data, which demonstrated that siRNA-mediated depletion of BTZ abrogates NMD elicited by tethered eIF4A3 [14]. Thus, in the context of tethered eIF4A3, the tight binding of MAGOH-Y14 is dispensable (although the faintly detectable binding of UPF3b may rescue some of the eIF4A3 function), but BTZ is required for NMD. It may appear from these data that a loss of the eIF4A3-BTZ pathway (Figure 3C, lanes 5–-) may have a stronger functional effect than the loss of the eIF4A3-UPF3b pathway (Figure 3C, lanes 3 and 4). However, because of the limitations of the tethering assay, the relevance of quantitative differences for NMD pathways remains to be explored in more depth.

We also used the complete set of mutant eIF4A3 proteins to analyze their ability to serve as EJC assembly platforms, both after completed splicing and at the step of C-complex formation (Figure 3D and 3E). Remarkably, eIF4A3 tolerates the lack of BTZ but not of MAGOH-Y14 or ATP binding to co-immunoprecipitate significant amounts of spliced mRNA (Figure 3D). Thus, the assembly of a stable (pre-)EJC does not require the incorporation of BTZ, while MAGOH and Y14 are indispensable, likely because they inhibit the ATPase activity of eIF4A3 and thus stabilize the complex [18]. The lack of ATP binding likely interferes with conformational changes as well as binding to MAGOH-Y14, explaining why these mutants fail to precipitate mRNA. When splicing is arrested before exon ligation, all eIF4A3 mutants immunoprecipitate less splicing intermediates than wild-type eIF4A3 does. When BTZ binding is abolished (lanes 5–8), three of the eIF4A3 mutants still co-precipitate significant amounts of RNA. To estimate the incorporation of the different mutants into either the spliceosomal C-complex (MINX GG) or the mature EJC (MINX), we compared the relative amounts of precipitated MINX and MINX GG for all eIF4A3 mutants. Interestingly, the class 1 mutants and the ATP binding mutants specifically impaired the stability of the EJC after the completion of splicing (up to 12-fold less relative MINX precipitation for class 1 mutants and up to 22-fold less for ATP binding mutants; Figure 3F), while the class 2 mutants do not affect the mature EJC (Figure 3F). Taken together, these data show that neither MAGOH-Y14 nor BTZ are completely required to support the loading of eIF4A3 during early splicing, and that MAGOH-Y14 and ATP (but not BTZ) are needed to stabilize the pre-EJC after splicing has been completed. Furthermore, MAGOH-Y14 are not required for NMD if eIF4A3 is stably associated with the RNA by tethering. Finally, the binding of BTZ to eIF4A3 is required for NMD, suggesting that this interaction helps establish a link to downstream NMD factors.

### Positioning of eIF4A3 during Splicing

**Figure 4:**
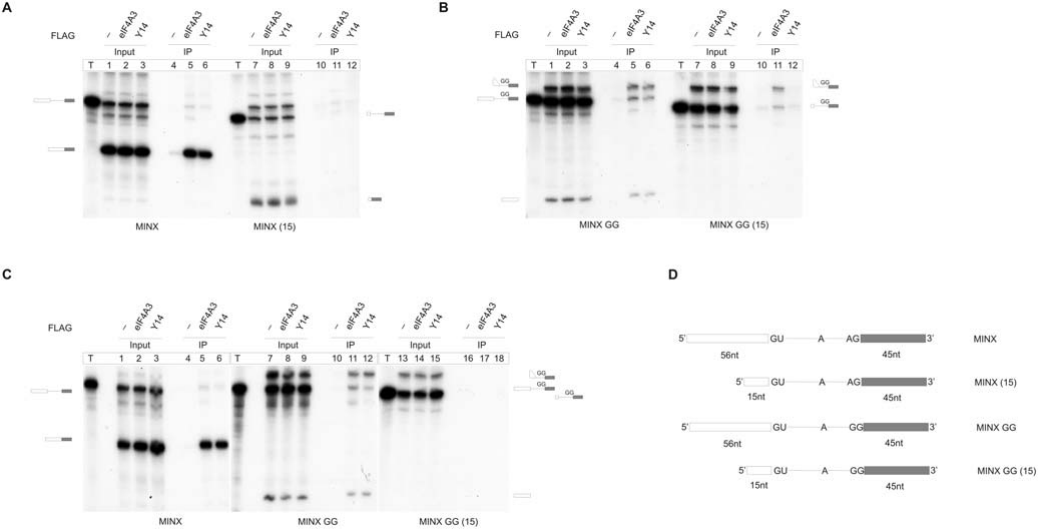
Successive formation of a trimeric pre-EJC. (A) Splicing reactions using MINX or MINX (15) as substrate RNAs were supplemented with FLAG-eIF4A3, FLAG-Y14, or FLAG-expressing extracts. Immunoprecipitations were done with FLAG affinity gel. Lanes with transcripts are labeled T. Twelve percent of the total input material was loaded in the input lanes. (B) Splicing reactions using MINX GG or MINX GG (15) as substrate RNAs were supplemented with FLAG-eIF4A3, FLAG-Y14, or FLAG-expressing extracts. Immunoprecipitations were done with FLAG affinity gel. Twelve percent of the total input material was loaded in the input lanes. (C) Splicing reactions using MINX, MINX GG, or MINX GG (15) as substrate RNAs were supplemented with FLAG-eIF4A3, FLAG-Y14, or FLAG-expressing extracts. Immunoprecipitations were done with FLAG affinity gel in the presence of 1% Empigen BB. Twelve percent of the total input material was loaded in the input lanes. (D) Schematic representation of the transcripts used in (A–C). Exons and introns are displayed as boxes and lines, respectively.

The data described above indicate that an ordered assembly hierarchy ensures the proper loading of EJCs during splicing and the function of EJCs in downstream processes such as NMD. Since EJC assembly from recombinant subunits on RNAs is not position-specific, whereas the spliceosome-mediated process is, we next aimed to identify the steps involved in EJC positioning by the spliceosome. To this end, we generated two additional MINX transcripts with shortened 5′ exons (15 nts), based on the constructs shown in Figure 1A and 1D with either the wild-type (AG) or the mutant GG 3′ splice site (MINX (15) and MINX GG (15); Figure 4D). Since the EJC is deposited onto the mRNA 20–24 nts upstream of the splice junction [4],[39],[40], our transcripts with the shortened 5′ exons lack physiologically defined EJC binding sites. We performed FLAG immunoprecipitations with all four transcripts after in vitro splicing with FLAG-tagged eIF4A3 and Y14. As shown before, eIF4A3 and Y14 precipitate the spliced MINX mRNA (Figure 4A, lanes 4–6). In contrast, spliced MINX (15) mRNA is not co-immunoprecipitated by either eIF4A3 or Y14, which confirms that this transcript lacks an EJC binding site (Figure 4A, lanes 10–12). The MINX GG transcript that does not undergo the second step of splicing is also co-immunoprecipitated with both proteins (Figure 4B, lanes 4–6), whereas, interestingly, the MINX GG (15) transcript, lacking an EJC binding site is co-precipitated with eIF4A3, but not Y14 (Figure 4B, compare lanes 11 and 12). eIF4A3 thus associates with early intermediates of the splicing reaction even when the RNA lacks an EJC binding site, but the association of eIF4A3 with the RNA is completely lost after the completion of splicing (Figure 4A, lane 11, see fully spliced product). This finding can be explained by two alternative models: (a) at an early step of EJC assembly, eIF4A3 binds to the RNA at a different position that is contained in MINX (15) and shifts later to the correct position; or (b) early binding of eIF4A3 to the RNA is indirect and mediated by additional factors, such as components of the spliceosome.

In order to distinguish between these models, we performed splicing reactions with the three transcripts that co-immunoprecipitate with eIF4A3 (MINX, MINX GG, and MINX GG (15)). After the completion of splicing, the samples were subjected to FLAG immunoprecipitation in the presence of the detergent Empigen BB (Figure 4C), which dissociates all but strong protein–protein and protein–RNA interactions. Under these conditions, eIF4A3 and Y14 co-immunoprecipitate only MINX and MINX GG RNAs that contain an EJC binding site (Figure 4C, lanes 5, 6, 11, and 12). In contrast, MINX GG (15) RNA is no longer co-immunoprecipitated by eIF4A3 under these stringent conditions (lane 17). Comparable results were obtained when the samples were UV-crosslinked before immunoprecipitation (Figures S2 and S3). This result demonstrates that the association of eIF4A3 with the MINX GG (15) RNA is mediated by interactions that are weaker than those within the fully assembled EJC. It also shows that Empigen BB-resistant EJCs can be detected within the C-complex, if an RNA binding site for the EJC is present. Y14 does not detectably associate with the MINX GG (15) RNA, even in the absence of a strong detergent (Figure 4B, lane 12). We suggest that MAGOH-Y14 binding may require a conformation of eIF4A3 ,which depends on its binding to the RNA EJC site. Taken together, these results indicate that during early phases of splicing, eIF4A3 interacts with the RNA indirectly, likely via spliceosomal proteins. Afterwards and possibly during one of the remodeling steps involved in spliceosome activation, eIF4A3 is directly deposited onto the RNA, which likely induces a conformational change that allows eIF4A3 to bind MAGOH-Y14, which may already be associated with the spliceosome.

### BTZ Associates with the EJC via eIF4A3

**Figure 5:**
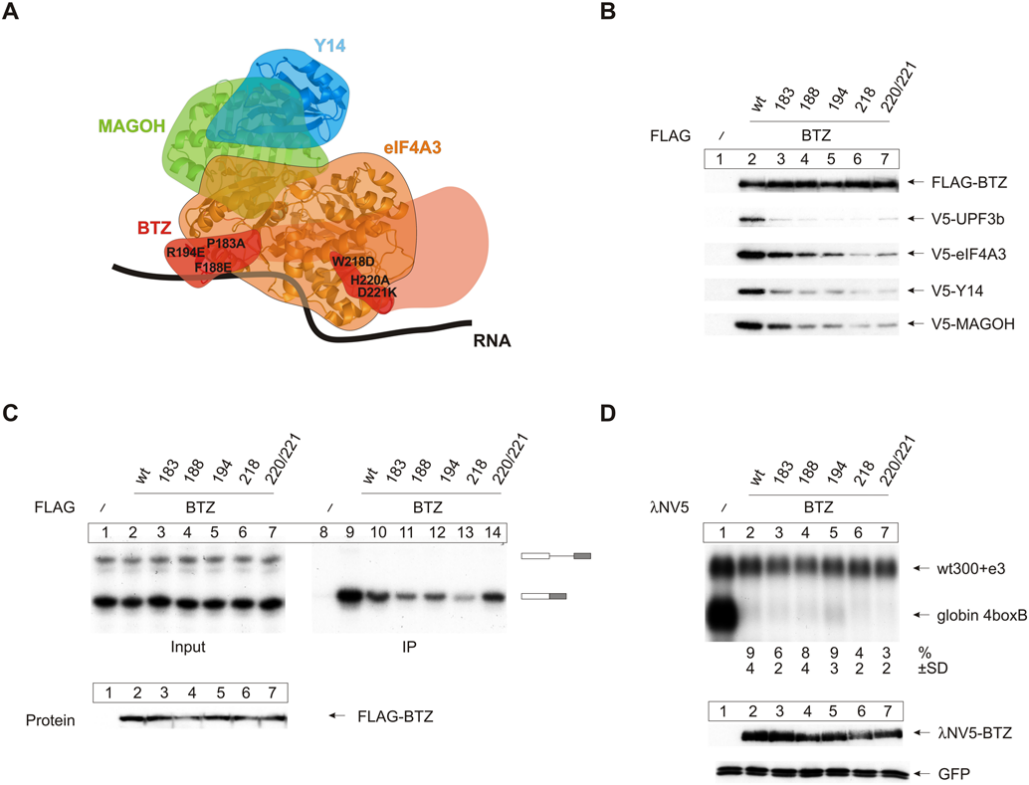
eIF4A3-binding deficient mutants of BTZ do not interact with the EJC, but elicit NMD when tethered to the RNA. (A) Positions of the BTZ mutations in the EJC as shown in Figure 2. (B) Immunoprecipitations were done from RNAse A-treated lysates of HeLa cells, which were transfected with FLAG-BTZ and FLAG-BTZ mutants or unfused FLAG as negative control together with V5-tagged UPF3b, eIF4A3, Y14, and MAGOH. Co-precipitated proteins were detected by immunoblotting using an anti V5 antibody. (C) Splicing reactions using MINX as substrate RNA were supplemented with extracts expressing the indicated FLAG-tagged BTZ mutants or unfused FLAG-tag as negative control. Reactions were immunoprecipitated with FLAG affinity gel. Twelve percent of the total input material was loaded in the input panel. (D) Northern blot analysis of total cytoplasmic RNA from HeLa cells that were transfected with expression plasmids for λNV5-tagged BTZ or mutants of BTZ together with the 4boxB reporter plasmid and the transfection control plasmid. Percentages (%) represent the mean of three independent experiments±standard deviations (SD). Bottom panel: expression levels of the tethered BTZ mutants were detected by immunoblot analysis with a V5 antibody. GFP served as an internal loading control.

Among the EJC core components, BTZ has unique characteristics: (1) it binds to the EJC after the completion of splicing (Figure 1), and (2) it is mostly cytoplasmic at steady state [41],[42]. Hence, BTZ may join the exported mRNP in the cytoplasm. We analyzed the features of BTZ that are required for its function during EJC biogenesis and NMD. Considering the crystal structure, we generated BTZ mutations to disrupt the binding of BTZ to eIF4A3 (Figure 5A). FLAG immunoprecipitation analysis of our BTZ mutants confirmed that their interaction with eIF4A3 (and indirectly with MAGOH-Y14) is indeed diminished albeit to different degrees (Figure 5B). Particularly the 218 mutant is severely impaired in its interaction with eIF4A3 (Figure 5B, compare lane 6 with lane 2). According to the crystal structure of the EJC core, BTZ associates with the EJC and the RNA via eIF4A3 [19],[20]. When tested experimentally, mutants of BTZ that show decreased eIF4A3 binding also co-immunoprecipitate reduced amounts of spliced MINX mRNA (Figure 5C). In excellent correlation with the protein co-immunoprecipitation data, the BTZ mutant 218 displays the weakest RNA association (Figure 5C, lane 13) which confirms that BTZ binds to the RNA mainly through its interaction with eIF4A3 [19],[20]. Compared to BTZ 218, the mutant 220/221 shows increased RNA association despite having only slightly stronger eIF4A3 binding. This suggests that residues of BTZ could also directly contribute to its interaction with RNA.

### BTZ Bridges the EJC to UPF1-Dependent RNA Degradation (NMD)

We next addressed the question of whether BTZ mutants that are not able to bind efficiently to the RNA through the EJC core proteins are able to activate NMD if bound to the RNA independently of the EJC. We thus performed BTZ tethering experiments. Remarkably, all of the BTZ mutants tested above show full activity in the NMD tethering assay (Figure 5D). These data indicate that the NMD-activating domain of BTZ is separable from the EJC-interacting domain. The residues of BTZ that stabilize the interaction with eIF4A3 and the other EJC proteins are not required for its NMD function when BTZ is independently tethered to the RNA. In contrast, recruitment of BTZ to a spliced mRNA requires its interaction with the EJC. These data identify BTZ as a functional bridging factor between the EJC and its NMD function.

**Figure 6:**
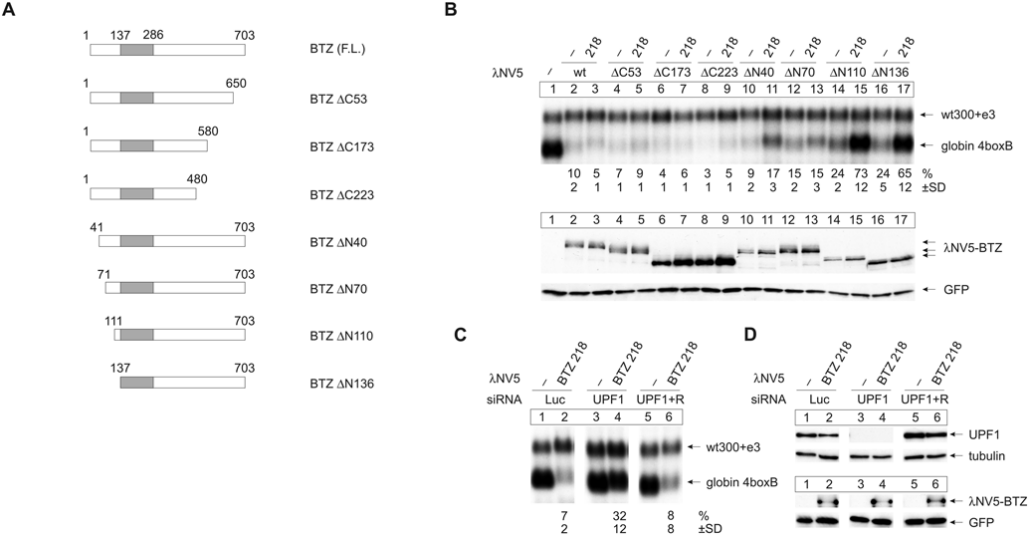
BTZ links the EJC with UPF1-dependent NMD. (A) Schematic representation of the BTZ deletion mutants used in (B). The SELOR domain is displayed as a gray box, the size of the respective N- or C-terminal deletion is indicated. (B) Northern blot analysis of total cytoplasmic RNA from HeLa cells that were transfected with expression plasmids for λNV5-tagged BTZ or deletion mutants of BTZ together with the 4boxB reporter plasmid and the transfection control plasmid. The presence of the 218 mutation in each mutant is indicated. Percentages (%) represent the mean of four independent experiments±standard deviations (SD). Bottom panel: expression levels of the tethered BTZ mutants were detected by immunoblot analysis with an anti-V5 antibody. GFP served as internal loading control. (C) Northern blot analysis of total cytoplasmic RNA from HeLa cells that were transfected with siRNAs targeting Luciferase (negative control) or UPF1. Thirty hours later, the cells were transfected with the expression plasmid for λNV5-tagged BTZ 218 together with the 4boxB reporter plasmid and the transfection control plasmid. The siRNA depletion was rescued by transfecting a siRNA-insensitive expression plasmid for UPF1. Percentages (%) represent the mean of four independent experiments±standard deviations (SD). (D) Upper panel: immunoblot analysis of UPF1 expression in lysates from cells used in (C) with a UPF1-specific antibody. Tubulin served as control for comparable loading. Lower panel: the expression of tethered BTZ 218 was detected by immunoblot analysis with an anti-V5 antibody. GFP served as internal loading control.

To identify the NMD effector domain of BTZ, we generated N- and C-terminal deletion mutants with or without the 218 mutation that reduces eIF4A3 binding (Figure 6A). Functional analysis in the NMD tethering assay shows that C-terminal deletions of up to 223 amino acids maintains the NMD activity of BTZ even in the presence of the 218 mutation (Figure 6B, lanes 4–9). Similarly, N-terminal deletions of 40 or 70 amino acids display only small effects (lanes 10–13). In contrast, deletion of 110 or 136 N-terminal amino acids reduces the activity of the respective mutant by approximately 2-fold. This loss of function is severely aggravated by the 218 mutation.

To test whether the activity of BTZ 218 is UPF1-dependent, a central hallmark of NMD, we depleted UPF1 in the tethering assay. UPF1 depletion abrogates the activity of the BTZ 218 mutant (Figure 6C), while the restoration of normal UPF1 levels by transfection of an expression vector for siRNA-insensitive UPF1 [14] completely restores NMD. This result proves that the observed UPF1 dependence is not caused by nonspecific or off-target effects. Taken together, these findings reveal that BTZ (likely amino acids (aas) 41–480) carries an EJC-independent NMD effector function and suggest that BTZ serves to bridge the EJC to the NMD machinery.

### BTZ Is Required for NMD

**Figure 7:**
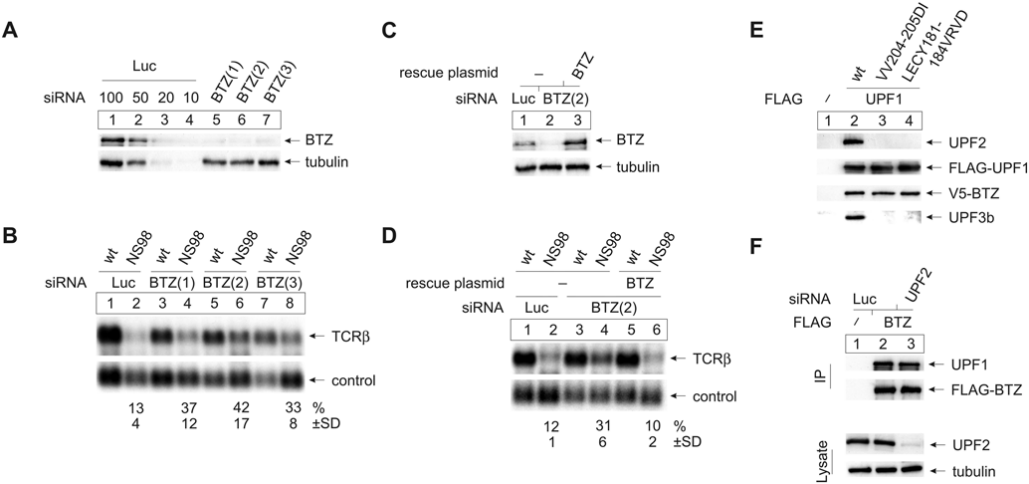
BTZ is required for efficient NMD and interacts with UPF1. (A) Immunoblot analysis for BTZ of protein lysates from HeLa cells transfected with Luciferase siRNA (negative control; lanes 1–4) or BTZ siRNAs (lanes 5–7). Dilutions corresponding to 50%, 20%, or 10% (lanes 2–4) of the initial protein amount (lane 1) of negative control, siRNA-transfected cells were loaded to assess the efficiency of the BTZ depletion. Reprobing with a tubulin-specific antibody was performed to control for loading. (B) Northern blot analysis of RNA from HeLa cells transfected with the indicated siRNAs and subsequently with the NMD reporter plasmids TCR-β wt or NS98 and a plasmid controlling for transfection efficiency. The numbers indicate changes in mRNA abundance±SD determined by analysis of four independent experiments. (C) Immunoblot analysis of protein lysates from HeLa cells transfected with siRNA targeting Luciferase (lane 1) or siRNA (2) targeting BTZ (lanes 2–3) as described in (A). To rescue the siRNA depletion, a siRNA-insensitive mutant of BTZ was co-transfected (lane 3). (D) Northern blot analysis of RNA from HeLa cells that were transfected with siRNA (lane 2). The NMD reporter plasmids TCR-β wt or NS98 and a transfection efficiency control were transfected together with a plasmid expressing a siRNA-insensitive variant of BTZ (lanes 5 and 6). The numbers indicate changes in mRNA abundance±SD determined by the analysis of four independent experiments. (E) Immunoprecipitations were done from RNAse A-treated lysates of HeLa cells that were transfected with FLAG-UPF1 and FLAG-UPF1 mutants together with V5-tagged BTZ. Co-precipitated proteins were detected by immunoblotting using UPF2, UPF3b, or anti V5 antibodies. (F) Immunoprecipitations were done from RNAse A-treated lysates of HeLa cells that were treated with siRNA and transfected with FLAG-BTZ. Co-precipitated proteins were detected by immunoblotting using an anti UPF1 antibodies. The depletion of UPF2 was assessed in the cell lysates with an UPF2 antibody. The membrane was reprobed with a tubulin antibody to control for loading.

Our data indicate that BTZ can activate UPF1-dependent NMD of a reporter mRNA in a tethering assay and predict that BTZ depletion should specifically inhibit NMD efficiency in a physiological model. Hence, we tested the role of BTZ in bona fide NMD by depleting endogenous BTZ protein in cells transfected with wild-type and nonsense-mutated TCR-β constructs as NMD reporters. We measured the efficiency of NMD in cells that were independently transfected with one out of three different siRNAs targeting BTZ (Figure S4). Depletion of BTZ by any of these three siRNAs (Figure 7A) significantly up-regulates the nonsense-mutated TCRβ reporter mRNA by approximately 3-fold (Figure 7B). The restoration of BTZ levels by a siRNA(2)-insensitive BTZ variant (Figure 7C) efficiently restored normal NMD activity (Figure 7D), confirming the specificity of the BTZ effect and excluding off-target effects.

If BTZ links the EJC to UPF1-dependent NMD, we reasoned that these two proteins should exit in one complex and that this complex should occur independently of the proteins UPF2 or UPF3. To test this hypothesis, we performed immunoprecipitations with FLAG-tagged UPF1 or mutants of UPF1 that lack interaction with UPF2 [43]. Whereas wild-type UPF1 co-immunoprecipitates UPF2, UPF3b, and BTZ (Figure 7E, lane 2), the two mutants precipitate only BTZ but no detectable quantities of either UPF2 or UPF3b (Figure 7E, lanes 3 and 4). Similarly, complex formation of BTZ and UPF1 was not compromised in cells depleted of UPF2 (Figure 7F). Hence, the interaction between BTZ and UPF1 is not mediated by UPF2 or UPF3b. These data demonstrate that BTZ can associate with the NMD protein UPF1 in a complex that is distinct from the complex containing UPF2 and UPF3b.

## Discussion

The core EJC consisting of the recombinant proteins eIF4A3, BTZ, MAGOH, and Y14 self-assembles in the presence of ATP on a RNA substrate in vitro [18], but this core EJC does not convey positional information that results from spliceosomal EJC assembly. The data provided by the crystal structure of the recombinant EJC core [19],[20] have provided a basis for our experimental strategy to analyze the assembly of the EJC by the spliceosome with an array of EJC protein mutants.

### Definition and Assembly of a Minimal Trimeric Pre-EJC

**Figure 8:**
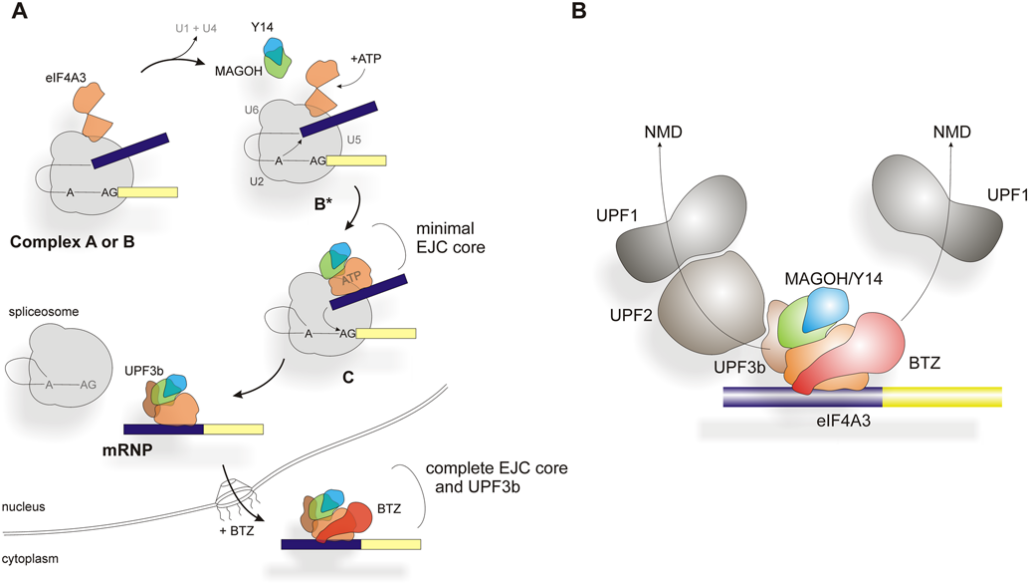
Ordered assembly of the EJC by the spliceosome. (A) Assembly pathway of the EJC. For details, see Discussion. (B) The exon junction complex recruits NMD activating proteins and combines different complexes leading to UPF1-dependent NMD. The model of the BTZ-dependent pathway is derived from the data presented in this work, whereas the model of the UPF3b-UPF2-UPF1-dependent pathway is derived from collective data of earlier publications [34],[35],[39],[49]. Details are explained in the Discussion.

We report here the definition of an ordered and hierarchical assembly pathway of the EJC during splicing (Figure 8A). This ordered assembly is initiated by the binding of eIF4A3 and MAGOH-Y14 to the spliceosome before exon ligation takes place (step 1). A candidate binding partner of eIF4A3 within the spliceosome may be the protein IBP160 (AQR) that has been suggested to recruit the EJC to the intron during splicing [44]. Considering the data shown in Figure 4 and the crystal structure of the EJC core [19],[20], we propose that the spliceosome introduces eIF4A3 to the RNA with an associated change of the conformation of this protein after ATP binding (step 2). This enables the binding of MAGOH-Y14 (step 3), completing the assembly of the first stable EJC intermediate that we refer to as minimal pre-EJC or trimeric pre-EJC. This assembly pathway also takes full account of both, a previous analysis of EJC assembly [24] and the analysis of purified spliceosomes and spliceosomal sub-complexes, where eIF4A3, MAGOH, and Y14 were identified in the splicing complex B, the activated spliceosome (B*), and splicing complex C [26]–[28].

Our data also suggest that the trimeric pre-EJC represents the platform that physiologically interacts with additional EJC components. The purification of spliced mRNPs devoid of detectable amounts of BTZ has been reported, suggesting that stable (pre-) EJCs without BTZ can occur under physiological conditions [45]. Interestingly, recombinant EJC proteins from bacteria fail to interact with the spliceosome, and these bacterially expressed proteins appear to assemble on the RNA during the second step of splicing [38]. The differences of the results obtained with bacterially expressed proteins [38] and EJC proteins from eukaryotic sources (this study and [26]) suggest that posttranslational modifications of EJC proteins in mammalian cells may be required for the association of EJC protein(s) with the spliceosome.

The spliceosome undergoes conformational and structural changes during its assembly and activation for splicing [26],[27]. How the suggested conformational change of eIF4A3 is achieved in this context remains an open question. The conformational change of eIF4A3 may be a consequence of the rearrangements that occur within the spliceosome when splicing proceeds. Because eIF4A3 is found in the activated spliceosome, eIF4A3 may adopt the closed, RNA-bound conformation during the transition from the activated spliceosome to splicing complex C. The results shown in Figures 2–4 strongly suggest that a significant fraction of eIF4A3 adopts the MAGOH-Y14–bound state before exon ligation. We thus propose that eIF4A3 assumes a closed confirmation and is stably locked onto the RNA in the C-complex upon binding of ATP and MAGOH-Y14.

### From the Trimeric Pre-EJC to the NMD-Competent Core EJC

BTZ and the NMD factor UPF3b bind to the trimeric pre-EJC after the mRNA is released from the spliceosome. Considering the predominant localization of BTZ in the cytoplasm [41],[42] and the fact that BTZ can join the trimeric pre-EJC after splicing (Figure 1F), this interaction may likely occur after nuclear export of the mRNP. BTZ is thus proposed to join the EJC at a later (possibly cytosolic) step of assembly. Interestingly, the interaction between the trimeric pre-EJC and BTZ depends on exposed surface residues of eIF4A3 including aa 178/179 that also stabilize the association of eIF4A3 with spliceosomal complexes before the second step of splicing (Figure 3D and 3E). This overlap indicates that BTZ and the spliceosome interact with the same region of eIF4A3, a finding that can explain why nuclear BTZ fails to enter spliceosomal complexes and why BTZ has not been found in purified spliceosomal complexes [26].

### Implications of EJC Assembly for NMD and Alternative NMD Pathways

Alternative pathways of NMD have previously been proposed to exist in mammals [14],[36],[43]. The corresponding EJC architectures and mechanistic aspects of the proposed alternative NMD pathways are not known so far.

The recruitment of BTZ to the minimal trimeric pre-EJC offers a biochemical basis for a UPF3b- and UPF2-independent NMD pathway, because BTZ can interact with UPF1 independently of UPF2 and UPF3b (Figure 7). Note that the degradation of some endogeneous NMD targets depends selectively on either BTZ or UPF2 [14]. Furthermore, the interaction between UPF2 and UPF1 is not strictly required for NMD [43], and UPF3b-independent NMD has been documented in cell lines and confirmed in humans with UPF3b null-mutations [36],[37]. We thus propose the existence of a BTZ-dependent NMD pathway that shares the requirement for UPF1 with the classical UPF3b-UPF2 pathway (Figure 8B).

The NMD-promoting properties of BTZ (Figures 5 and 6) are important for the understanding of the functions of the other EJC proteins eIF4A3 and MAGOH-Y14. EJC-independent BTZ recruitment (by tethering) is sufficient to induce reporter mRNA degradation, and MAGOH-Y14 are not required under these conditions. BTZ also displays some pre-mRNA binding capacity (Figure 1) [38], which may function to destabilize a subset of unspliced mRNAs. However, under physiological conditions, recruitment of BTZ to an RNA is strictly EJC-dependent, which secures the specificity of the NMD pathway for spliced and translated mRNAs that retain an EJC. This EJC specificity is also maintained in the “classical” NMD pathway that depends on the interaction between the EJC and UPF3b, and subsequently UPF2 and UPF1 [35],[46]–[49].

Physiologically, the role of alternative NMD pathways has not yet been defined. It is possible that these pathways are complementary or partly redundant to ensure maximal efficiency of quality control to safely eliminate otherwise detrimental faulty transcripts [50],[51]. Alternatively, distinct NMD pathways may act, e.g., in different tissues, at different times of development, or at different times of the cell cycle, enabling discriminating control over the expression of NMD targets, especially physiological endogenous transcripts.

## Materials and Methods

### Plasmids

Plasmid constructs β-globin wild-type, -NS 39, -4boxB, -6MS2, pCI-λNV5, pCI-MS2, pCI-FLAG, pCI-V5, the transfection control (wt300+e3), and expression vectors for Y14, MAGOH, eIF4A3, BTZ, and UPF3b were described previously [14],[15]. Full-length PYM (WIBG) and CBP80 cDNAs were obtained by reverse transcription (RT)-PCR using total HeLa cell RNA and inserted into pCI-neo FLAG. Mutants of MAGOH were generated by site-directed mutagenesis or described previously [14]; mutants of eIF4A3 and BTZ were generated by site-directed mutagenesis. MINX [52] was cloned EcoRI/BamHI into pGEM4. MINX GG, MINX (15), MINX GG (15), and MINX Δi were generated by PCR-mutagenesis and inserted into pGEM4. All constructs were verified by DNA sequencing.

### Cell Culture, Plasmid Transfections, and siRNA Transfections

HeLa cells were grown and transfected with plasmid DNA or siRNA as previously described [14]. For tethering experiments, 0.8 μg of MS2- or λNV5-fusion construct, 0.5 μg of the control plasmid (wt300+e3), 2 μg of the 4boxB or 6MS2 reporter vector, and 0.2 μg of a GFP expression plasmid were transfected. For transfections of β-globin wild-type or NS39, we used 1 μg of pCI-wt or NS39, 0.5 μg of the control plasmid, and 0.3 μg of the GFP expression vector. Transfections for immunoprecipitations were done with 3–4 μg of the FLAG-expression plasmid and 1 μg of GFP- and 0.8 μg V5-expression plasmids. GripTite 293 MSR cells (Invitrogen) were transfected with 6–8 μg of FLAG expression plasmids and 2 μg GFP expression vector in 10-cm cell culture dishes. siRNA transfections were done as previously described [14]. DNA target sequences for BTZ siRNAs were: (1) AACAUUCGCUCAGCUCAUAAU [16]; (2) GGUGGAUUCUAGUACAAGUTT (Ambion siRNA s22391); and (3) CCACCUCAGUUUAACCGGATT (Ambion siRNA s22392).

Complementation of the UPF1 depletion was done with the siRNA insensitive FLAG-UPF1^R^ expression plasmid described before [14]. The complementation of the BTZ depletion was done analogously to the UPF1 complementation by transfecting 0.3 μg of an expression plasmid for a siRNA-insensitive FLAG-BTZ^R^.

### RNA Extraction and Analysis

Total cytoplasmic RNA or total RNA were analyzed by Northern blotting [14]. Signals were quantified in a FLA-3000 fluorescent image analyzer (Raytest). Percentages were calculated as described [14],[15].

### Protein Extraction, Immunoblot Analysis, and Immunoprecipitation

Immunoblot analysis was performed using 10–30 μg of cytoplasmic or whole-cell extracts. After SDS-PAGE, proteins were transferred to a PVDF-membrane. Blocking was done with 5% non-fat skimmed milk in TBS-Tween (0.1%).

FLAG complexes were immunoprecipitated from RNase A-treated HeLa cell lysates (20 μg/ml) with M2 anti-FLAG agarose (Sigma) at 4°C for 1–2 h in lysis buffer (50 mM Tris [pH 7.2], 150 mM NaCl, 1 mM EDTA, 0.5 mM PMSF, 0.5% Triton X-100, + complete [Roche] protease inhibitor). Beads were washed four times in lysis buffer without protease inhibitors. Precipitated complexes were eluted with SDS-sample buffer and analyzed by immunoblotting.

### In Vitro Transcription, In Vitro Splicing, and RNP Immunoprecipitation

Capped transcripts were generated by in vitro transcription with SP6 RNA polymerase in the presence of m7GpppG cap analog (Promega). In vitro splicing reactions were performed for 2 h in HeLa cell nuclear extract (CIL Biotech) that was supplemented with 293 whole-cell extracts from cells transfected with different FLAG-tagged EJC proteins [53]. To prepare HEK293 whole-cell extracts, cells from a single 10-cm dish were resuspended in 200–300 μl of Buffer E (20 mM Hepes-KCl, pH 7.9, 100 mM KCl, 0.2 mM EDTA, 10% glycerol, 1 mM DTT) and lysed by sonication [53]. FLAG-immunoprecipitations of RNPs were performed with FLAG-M2 affinity gel in mRNP IP buffer (20 mM HEPES KOH, pH 7.9, 200 mM NaCl, 2 mM MgCl_2_, 0.2% Triton X-100, 0.1% NP-40, 0.05% Na-Deoxycholate). Empigen BB was used at a concentration of 1% during the binding and washing steps (Figure 4C). RNAs were recovered by TRI reagent extraction and isopropanol precipitation, a proteinase K treatment was included in the experiment shown in Figure 4C. The RNA was analyzed by denaturing PAGE. Twelve to fifteen percent of the total input material was loaded in the input panels (8% in Figure 1E).

### Antibodies

Antibodies to BTZ [42] and UPF2 [34] were kindly provided by Catherine Tomasetto and Jens Lykke-Andersen, respectively. Antibodies to UPF1 were raised using a GST-tagged N-terminal fragment (400 aa) of UPF1, antibodies to MS2 were raised using recombinant GST-tagged full length MS2, antibodies to UPF3b were raised using a recombinant GST-tagged C-terminal fragment of UPF3b. The antibody to GFP was from Abcam, the FLAG- and V5-antibodies were from Sigma.

## Supporting Information

Figure S1 **Splicing-independent BTZ binding to minimal EJC cores consisting of eIF4A3 and MAGOH-Y14,** (A) Splicing reactions using MINX as substrate RNA were supplemented with recombinant, Strep-tagged EJC proteins as indicated. Pulldowns were performed with StrepTactin spin columns. Positions of the unspliced transcript and the spliced product are displayed schematically. (B) Nuclear extracts were supplemented with the indicated recombinant proteins and an intronless MINX transcript (MINX i). Pulldowns were performed as in (A). (C) Comassie Brilliant Blue stained gel of the recombinant proteins used in (A) and (B).
(0.87 MB TIF)

Figure S2 **Crosslinking does not stabilize interactions between the pre-EJC and the short 5′ splicing substrate.** Splicing reactions using MINX, MINX GG, or MINX GG (15) as substrate RNAs were supplemented with FLAG-eIF4A3, FLAG-Y14, or FLAG-expressing extracts as described in Figure 4. Reactions were UV-crosslinked and immunoprecipitations were done with FLAG affinity gel in the presence of 1% Empigen BB. Crosslinked proteins were digested with Proteinase K after immunoprecipitation. Twelve percent of the total input material was loaded in the input lanes.
(3.23 MB TIF)

Figure S3 **Comparison of immunoprecipitation conditions with or without crosslinking.** Splicing reactions using MINX GG as substrate RNAs were supplemented with FLAG-eIF4A3, FLAG-Y14, or FLAG-expressing extracts. Reactions were divided into two parts: one was not treated (lanes 7–9) the other was UV-crosslinked (lanes 4–6). Immunoprecipitations were done with FLAG affinity gel in the presence of 1% Empigen BB. Proteins were digested with Proteinase K after immunoprecipitation and RNA extracted with TRI reagent. Ten percent of the total input material was loaded in the input lanes.
(1.58 MB TIF)

Figure S4 **Establishing the knockdown of BTZ.** Quantitative real-time PCR (qRT-PCR) measurement of BTZ mRNA levels after transfection of siRNA targeting Luciferase (Luc) or BTZ (1; 2; 3).
(0.08 MB TIF)

Table S1 **MAGOH mutants used in this study.** The table summarizes the names and respective mutations of the MAGOH mutants used in this study. Previously published mutations are indicated.
(0.03 MB DOC)

## Abbreviations

aas: amino acids
EJC: exon junction complex
mRNA: messenger RNA
NMD: nonsense-mediated mRNA decay
nts: nucleotides
UTR: untranslated region
