# Supplementary figures and images for: The Hierarchy of Exon-Junction Complex Assembly by the Spliceosome Explains Key Features of Mammalian Nonsense-Mediated mRNA Decay
Source: PLoS Biol. 2009 May 26;7(5):e1000120. doi: 10.1371/journal.pbio.1000120 (PMC2682485; doi:10.1371/journal.pbio.1000120)

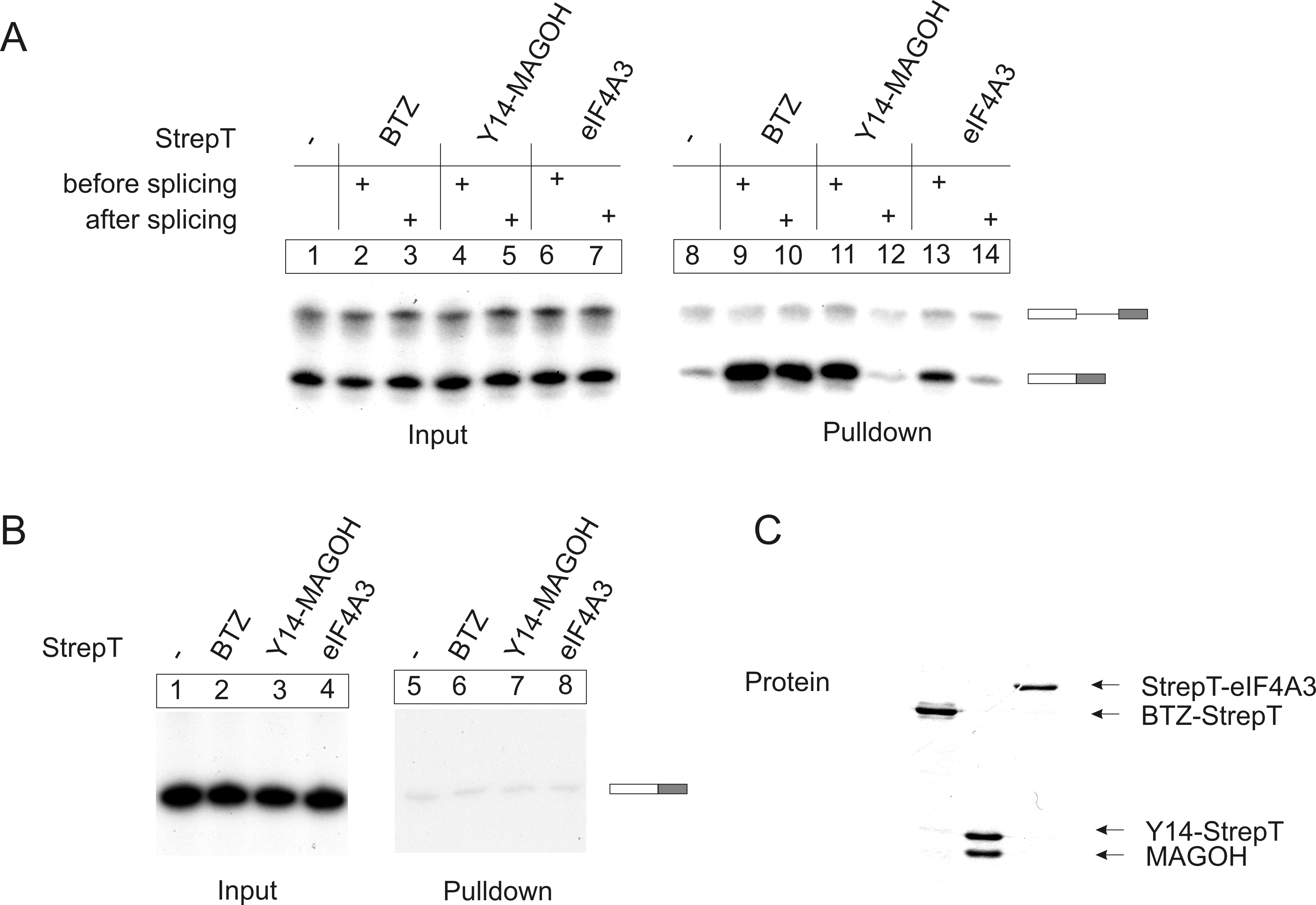

Supplement: Figure S1 — Splicing-independent BTZ binding to minimal EJC cores consisting of eIF4A3 and MAGOH-Y14, (A) Splicing reactions using MINX as substrate RNA were supplemented with recombinant, Strep-tagged EJC proteins as indicated. Pulldowns were performed with StrepTactin spin columns. Positions of the unspliced transcript and the spliced product are displayed schematically. (B) Nuclear extracts were supplemented with the indicated recombinant proteins and an intronless MINX transcript (MINX i). Pulldowns were performed as in (A). (C) Comassie Brilliant Blue stained gel of the recombinant proteins used in (A) and (B). (0.87 MB TIF) [file pbio.1000120.s001.tif]

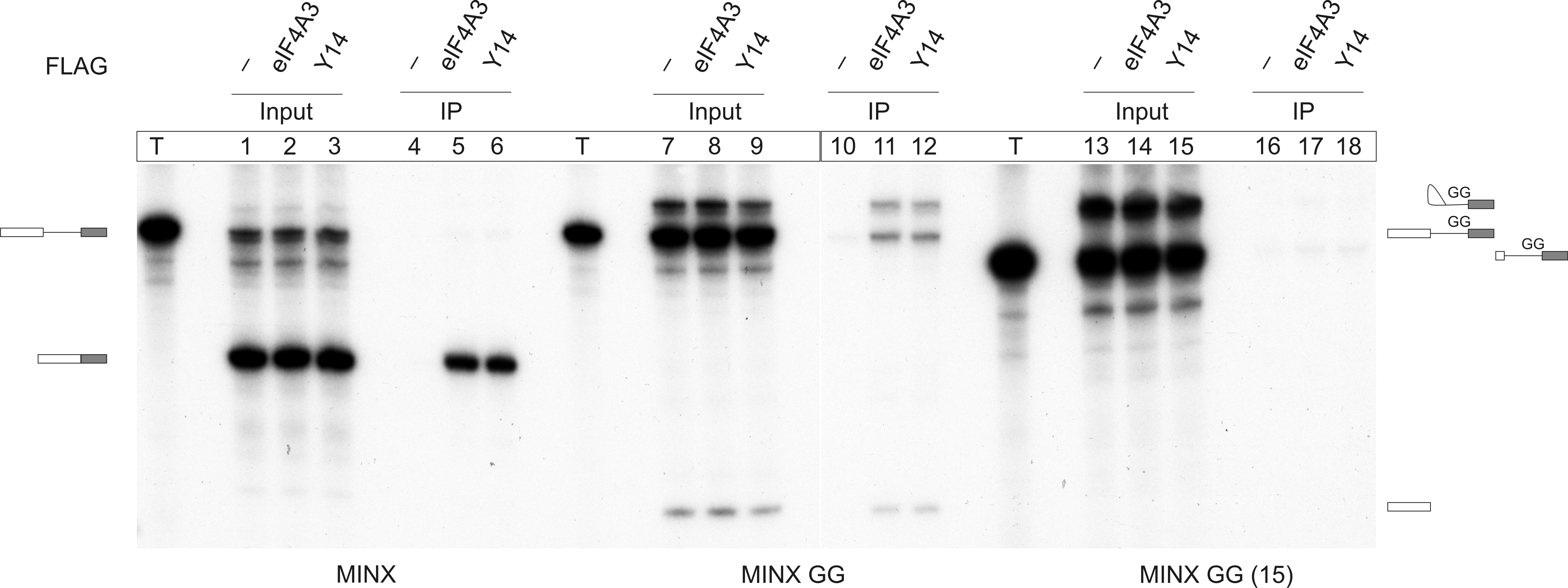

Supplement: Figure S2 — Crosslinking does not stabilize interactions between the pre-EJC and the short 5′ splicing substrate. Splicing reactions using MINX, MINX GG, or MINX GG (15) as substrate RNAs were supplemented with FLAG-eIF4A3, FLAG-Y14, or FLAG-expressing extracts as described in Figure 4. Reactions were UV-crosslinked and immunoprecipitations were done with FLAG affinity gel in the presence of 1% Empigen BB. Crosslinked proteins were digested with Proteinase K after immunoprecipitation. Twelve percent of the total input material was loaded in the input lanes. (3.23 MB TIF) [file pbio.1000120.s002.tif]

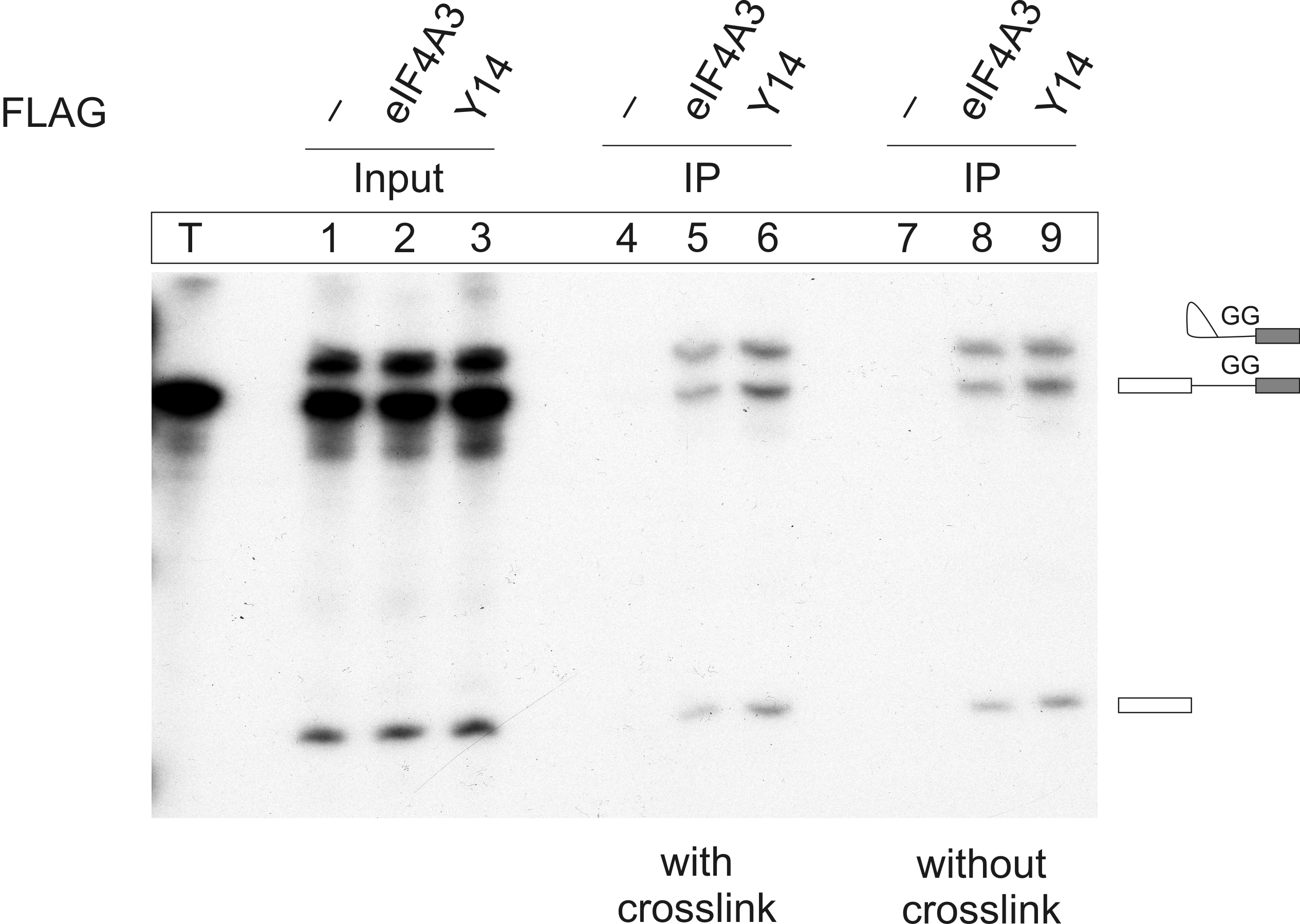

Supplement: Figure S3 — Comparison of immunoprecipitation conditions with or without crosslinking. Splicing reactions using MINX GG as substrate RNAs were supplemented with FLAG-eIF4A3, FLAG-Y14, or FLAG-expressing extracts. Reactions were divided into two parts: one was not treated (lanes 7–9) the other was UV-crosslinked (lanes 4–6). Immunoprecipitations were done with FLAG affinity gel in the presence of 1% Empigen BB. Proteins were digested with Proteinase K after immunoprecipitation and RNA extracted with TRI reagent. Ten percent of the total input material was loaded in the input lanes. (1.58 MB TIF) [file pbio.1000120.s003.tif]

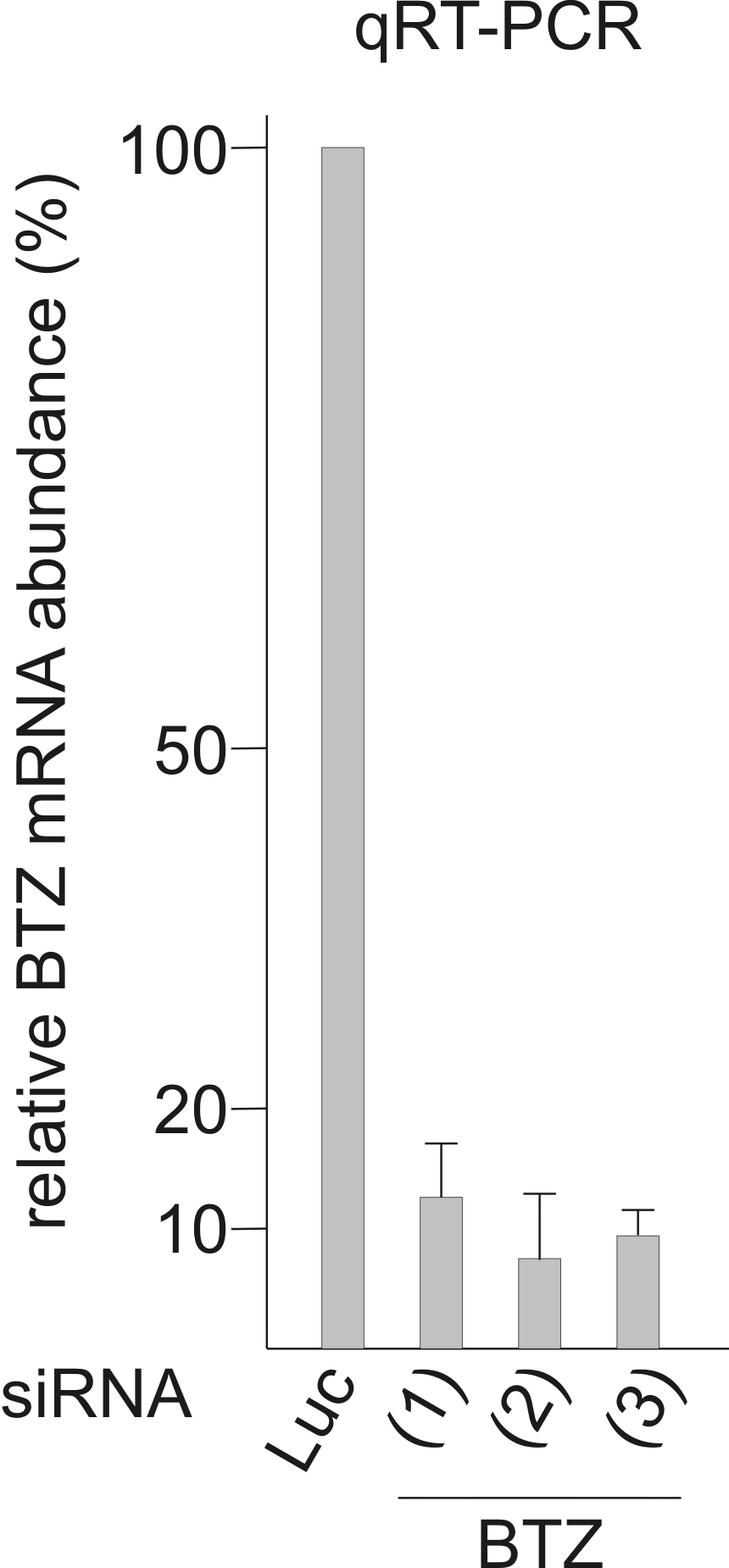

Supplement: Figure S4 — Establishing the knockdown of BTZ. Quantitative real-time PCR (qRT-PCR) measurement of BTZ mRNA levels after transfection of siRNA targeting Luciferase (Luc) or BTZ (1; 2; 3). (0.08 MB TIF) [file pbio.1000120.s004.tif]
