# Supplementary material for: The Hierarchy of Exon-Junction Complex Assembly by the Spliceosome Explains Key Features of Mammalian Nonsense-Mediated mRNA Decay
Source: PLoS Biol. 2009 May 26;7(5):e1000120. doi: 10.1371/journal.pbio.1000120 (PMC2682485; doi:10.1371/journal.pbio.1000120)
Supplement: Table S1 — MAGOH mutants used in this study. The table summarizes the names and respective mutations of the MAGOH mutants used in this study. Previously published mutations are indicated. (0.03 MB DOC) [file pbio.1000120.s005.doc]

| Name | Mutation | Reference |
| --- | --- | --- |
| 68 | E68R | this report |
| 72/73 | E72R/D73K | Gehring et al., 2005 |
| 117 | E117R | this report |
| 66/68 | D66R/E68R | Fribourg et al., 2003 |
| 16/17 | K16E/F17A | Fribourg et al., 2003 |
| 20 | E20R | this report |
| 39/40 | N39E/Y40A | this report |
| 41/42 | K41D/N42A | Gehring et al., 2005 |
| 130/134 | K130E/F134A | Fribourg et al., 2003 |
